# Human Tolerance in Romanian Brown Bears (*Ursus arctos*): Access to Human Food Sources Can Lead to Changes in Bear Behaviour

**DOI:** 10.1002/ece3.74359

**Published:** 2026-11-09

**Authors:** László Gál, Írisz‐Barbara Dobos, András Liker, István Elek Maák

**Affiliations:** ^1^ Hungarian Department of Biology and Ecology Babeş‐Bolyai University Cluj‐Napoca Romania; ^2^ Laboratory for Sociobiology and Insect Ecology, Centre for Systems Biology, Biodiversity and Bioresources Faculty of Biology and Geology, Babeș‐Bolyai University Cluj‐Napoca Romania; ^3^ HUN‐REN‐PE Evolutionary Ecology Research Group University of Pannonia Veszprém Hungary; ^4^ Behavioral Ecology Research Group, Center for Natural Sciences University of Pannonia Veszprém Hungary; ^5^ Department of Ecology, Faculty of Science and Informatics University of Szeged Szeged Hungary; ^6^ Laboratory of Social and Myrmecophilous Insects, Museum and Institute of Zoology Polish Academy of Sciences Warsaw Poland

**Keywords:** electric fence, fearful behaviours, food conditioning, garbage, increased human tolerance, insufficient protective measures

## Abstract

Tolerance to humans in wild animals can lead to several conflict situations and contribute to a generally negative perception of these animals among people. When a wild animal becomes reliant on human‐derived food, dangerous situations can arise, posing risks to both animals and humans. The brown bear ( *Ursus arctos* ) is a controversial species in Romania, as it is protected but also responsible for incidents with livestock, crops, or even humans. In our study, we aimed to identify the factors that facilitate bear visits to the town of Băile Tușnad and assess their effects on bear behaviours associated with increased tolerance of humans. Using city surveillance cameras, we analysed bear behaviour in relation to the use of electric fences and the presence of garbage scattered around the bins, seeking for a better understanding of which factors should be mitigated or adjusted to improve the safety of both bears and citizens. Our results supported our hypotheses that frequent bear visits to garbage container islands are linked to the availability of garbage and insufficient protective measures, such as non‐activated electric fences. As the frequency of bear visits increased, we observed a decrease in fearful behaviours, such as hesitation before entering the container islands or scanning their surroundings while foraging. This suggests that the bears of Băile Tușnad, in addition to showing signs of food conditioning, were also exhibiting increased tolerance to the urban environment, both of which could serve as precursors of a more persistent tolerance to human activity in the long term.

## Graphical abstract

In our study, we identified deficiencies in waste management that certain bears exploit to access easy food, leading to artificial food conditioning and increased tolerance to humans. Our analysis of urban surveillance footage revealed frequent bear visits to garbage bin islands, linked to improper use of protective measures, such as non‐activated electric fences, resulting in behavioural changes characterised by reduced fearfulness over time. These findings show the importance of effective attractant management and consistent preventive practices to mitigate human–bear conflicts.

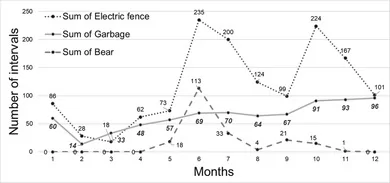

The tolerance of wild animals to humans is a globally recognised phenomenon and is often associated with increasing anthropogenic presence in natural landscapes, which can lead to potentially negative ecological and behavioural consequences (Wheat and Wilmers 2016). Habituation is a non‐associative form of learning in which an animal's response to a repeated stimulus diminishes over time. It allows animals to avoid costly reactions when repeated fearful responses to a non‐threatening stimulus no longer provide any benefit (Blumstein 2016). A wild animal's natural fear response plays a crucial role in its survival, as reacting appropriately to predators increases life expectancy (Christensen et al. 2006). However, risk‐avoidance behaviours, such as fleeing, are energetically costly, leading animals to minimise these costs while maximising survival (Moen et al. 2019). When an animal repeatedly encounters a potentially threatening situation without experiencing negative consequences or reinforcement, it may gradually withhold its escape response and begin to habituate (Christensen et al. 2006).

Understanding the behavioural mechanisms underlying tolerance is crucial for addressing situations where it occurs (Liker et al. 2026). Increased tolerance to humans has historically played a significant role in shaping animal‐human relationships. For instance, domestication was, at least initially, facilitated by the gradual habituation of wild animals to human presence (Clutton‐Brock 1992). Similarly, animal training often employs gradual exposure to novel stimuli, which accelerates habituation and results in more pronounced behavioural changes (Christensen et al. 2006, Thompson 2009). Understanding this process is important for wildlife management to prevent scenarios and conditions that lead to the loss of fear responses towards humans.

In wild animals, fear of humans is an adaptive response that evolved through historical negative interactions (e.g., hunting practices, removal of conflictual individuals) and remains prevalent (Brubaker and Coss 2015). For instance, in bears, natural selection favoured the survival of fearful individuals, as bold behaviour increased the chance of human encounters and subsequent mortality. Although boldness once allowed bears to explore new areas and resources, these individuals were often removed from populations, leading to a predominance of more cautious individuals over evolutionary time (Herrero 2018). This selection pressure likely explains why the default behavioural response of bears is to flee rather than engage unless provoked (Herrero 2018). Similar behavioural responses to repeated human exposure have been documented across a wide range of taxa, including large carnivores, ungulates and marine mammals, highlighting that habituation is a general process rather than one specific to bears (Bejder et al. 2009, Haidt et al. 2018, Dsouza et al. 2025, Liker et al. 2026).

The widespread expansion of human presence and activities into natural areas increases the frequency of bear‐human interactions (Morales‐González et al. 2020). In response, bears often adopt human‐avoidance strategies, such as shifting to nocturnal activity to minimise encounters with humans. However, exploiting anthropogenic food resources involves ecological trade‐offs. Although food rewards may be substantial, bears often restrict their activity to periods of low human activity, primarily during the night, thereby reducing the temporal window available for exploiting anthropogenic food resources. The ecological consequences of this behavioural shift depend on the type of food resource available; for example, nocturnal foraging may increase foraging success in some natural systems (Reimchen 1998), whereas in urban environments it primarily reflects a strategy to minimise the risk of encounters with humans (Ordiz et al. 2014; Morales‐González et al. 2020). In contrast, bears tolerant to humans can exploit new food sources and habitats within human‐dominated landscapes (Wheat and Wilmers 2016). When the benefits of reducing antipredator responses outweigh the associated risks, bears can become tolerant to human proximity and exploit the advantages of human‐dominated environments (Herrero et al. 2005; Bateman and Fleming 2012). However, bears that become overly reliant on anthropogenic food sources (food‐conditioned) are more likely to approach human settlements in search of food, increasing the risk of lethal removal (e.g., hunting, poaching, intervention activity), injury, or death from vehicle or train collisions (Spalona 2012; Elfström, Zedrosser, Jerina, et al. 2014). Allowing bears access to anthropogenic food sources poses significant risks for both bears and humans. For instance, it can create an ecological trap, where bears select areas based on environmental cues that appear beneficial but ultimately lead to negative outcomes (Morales‐González et al. 2020). Additionally, feeding bears for observational or touristic purposes may condition them to associate human presence with food sources, particularly when such feeding sites are located in proximity to human settlements (Penteriani et al. 2017).

Although human‐bear conflicts related to garbage foraging and food conditioning are well‐documented in North America, such studies in European urban contexts remain scarce. The brown bear ( *Ursus arctos* ) is simultaneously a conservation flagship species, a major tourist attraction, and a source of human‐wildlife conflict in Romania and elsewhere. By the twentieth century, this emblematic species had disappeared from many European countries due to habitat loss and persecution (Penteriani et al. 2020). However, in countries like Romania, conservation efforts and legal protections in recent decades allowed bear populations to recover and maintain their ecological role as opportunistic omnivores (Bereczky et al. 2011; Stăncioiu et al. 2019). Despite these efforts, the natural behaviour of brown bears has been significantly altered by inadequate waste management and deliberate feeding for touristic purposes, leading to increased tolerance to humans and an association of human presence with easy access to food sources and other advantages of human proximity (Elfström, Zedrosser, et al. 2014; Penteriani et al. 2017).

In this study, we aimed to examine year‐round patterns of bears' visits to urban feeding sites (two garbage container islands) and hypothesised that repeated visits may represent early stages of food conditioning and increased tolerance to humans. We also hypothesised that garbage availability and the presence of electric fences influence bear foraging behaviour, with potential individual variation (Bereczky 2016). To address these hypotheses, we analysed several behavioural metrics, including (a) hesitation, (b) vigilance, (c) foraging duration, and (d) total visit duration. We predicted that with repeated visits (i) hesitation and (ii) vigilance times would decrease, while (iii) foraging duration and (iv) total visit duration would increase, indicating a higher level of tolerance. Additionally, we predicted that (v) garbage availability would positively influence bear activity, while (vi) the presence of electric fences would reduce it.

## 1 Materials and Methods

### 1.1 Study Area and Methodology

Romania contains one of the largest remaining continuous natural landscapes in Europe, with 66,303 km^2^ of the Carpathian Mountains within its borders. Traditional land use practices are still prevalent throughout this region, resulting in a mosaic of small‐scale, diverse agricultural practices, including orchards, livestock pastures, hay meadows, and forests. Dense shrub vegetation is also common along riverbanks. The bear population in Romania is estimated to exceed 6000 individuals and is considered to be increasing. However, current official estimates are not based on systematic surveys and are generally considered to be overestimates (Pop et al. 2012; Popescu et al. 2017).

The study was conducted year‐round between 01 October 2021 and 30 September 2022 at two garbage container islands (GC1 and GC2) in Băile Tușnad (46°08′49″ N, 25°51′38″ E), the smallest town in Romania by population and a popular tourist destination. The town is located in the Olt River valley at an altitude of 650 m, surrounded by the dense forests of Harghita and Bodoc Mountains, and is located in the middle of an ecological corridor (Figure 1). The surrounding forests are predominantly composed of European silver fir ( *Abies alba* ) and European beech ( *Fagus sylvatica* ), and provide abundant natural food sources for bears, including soft mast (e.g., raspberries, blackberries (*Rubus* species) and wild strawberries ( *Fragaria vesca* )), hard mast (mainly beechnuts), ant colonies, and a variety of herbaceous plants. Bears also seasonally exploit nearby agricultural areas (e.g., maize, wheat, oats), gardens, and fruit trees, particularly apple and plum.

**FIGURE 1:**
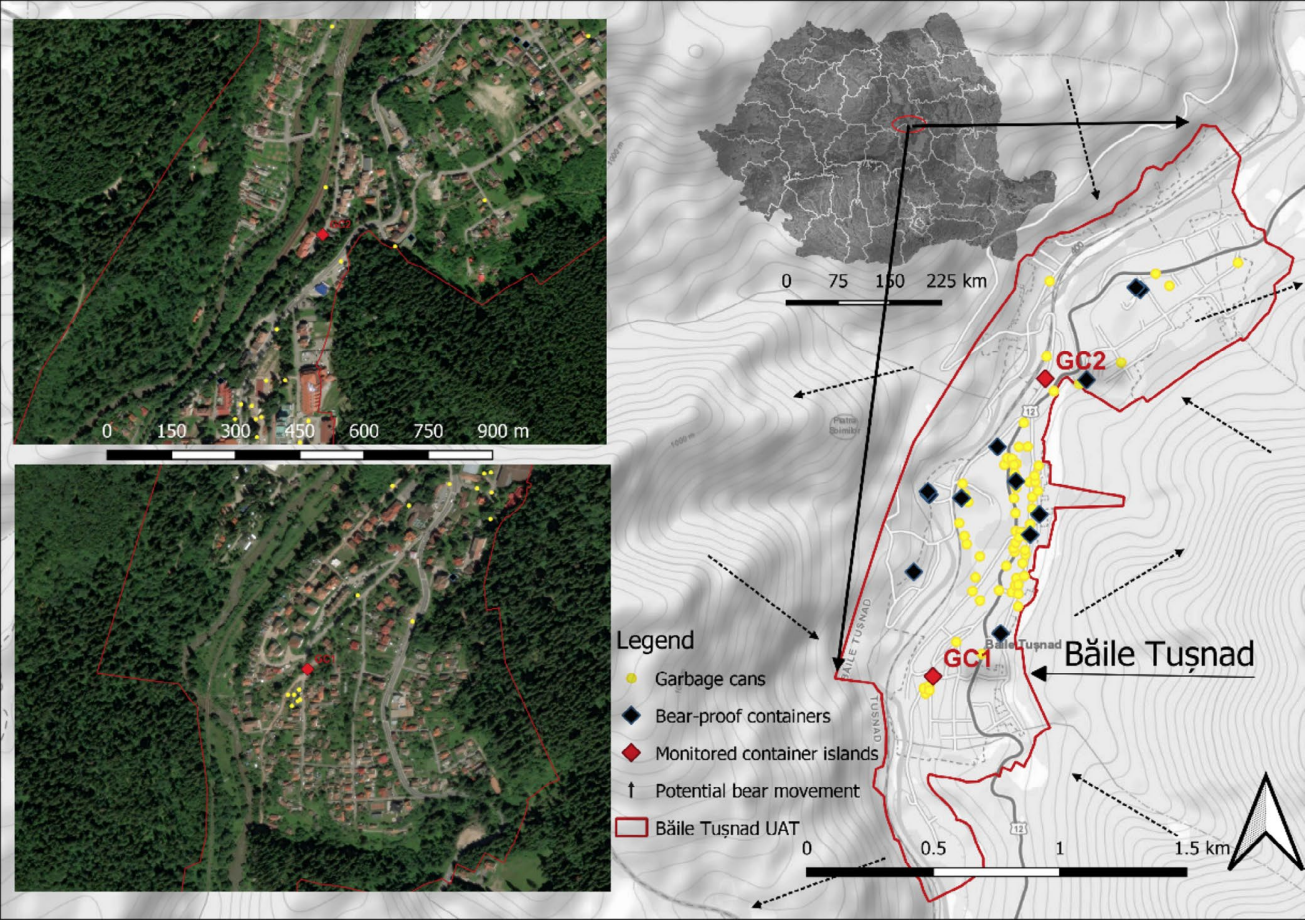
Map (right panel) shows the location of the study area within Romania, as well as the surrounding topography, the town boundary of Băile Tușnad, and the locations of the two monitored garbage container islands (GC1 and GC2; red rhombuses). Bear‐proof garbage containers are indicated by black rhombuses, whereas all other garbage receptacles are shown as yellow dots. High‐resolution satellite imagery (left panels) illustrates the immediate surroundings of GC1 (bottom) and GC2 (top). Black arrows indicate the hypothesised directions of bear movement into the urban area. The surrounding forests form a continuous ecological corridor that provides unrestricted access to the town from multiple directions, highlighting the high landscape connectivity of the study area.

The two garbage container islands were situated within the urban area. GC1 was located 197 m from the eastern forest edge and 187 m from the western forest edge, whereas GC2 was located 58 m from the eastern forest edge and 216 m from the western forest edge (Figure 1). The forest edge does not necessarily coincide with the administrative boundary of the town. Each garbage container island consisted of six garbage containers (1100 L in total) enclosed within bear‐proof, heavy‐metal cases with lid handles, preventing bears from directly accessing the waste inside. The containers were arranged in two parallel rows of three containers separated by approximately 380 cm between the two rows. Both garbage container islands were located close to human infrastructure: GC1 was situated 30 m from the nearest residential building, whereas GC2 was located 10 m from the nearest garage, 20 m from the railway station and 80 m from the nearest house. Both sites were surrounded by three‐wire electric fences placed at an average of 15 cm from the containers. Access by people required uncoupling the fence wires. Although the fences were intended to remain active continuously, they were often left open during daytime and occasionally overnight. Household waste, including food remains, was collected at both sites, providing bears with access to anthropogenic food whenever the electric fences were not secured.

Behind each garbage container island was a densely vegetated area consisting of shrubs, trees and tall vegetation that was not illuminated by street lights. We defined these areas as “safe zones” because bears frequently retreated there with garbage collected from the containers and consumed it under cover. Garbage remains were regularly observed within these areas, suggesting repeated use as sheltered foraging locations. In front of each garbage island was a road with adjacent parking spaces (Figure S1). Garbage containers were emptied once per week. However, waste frequently accumulated outside the containers between collection events, allowing bears to access food without opening the containers.

### 1.2 Data Collection and Camera Footage Analysis

Two town surveillance cameras overlooking GC1 and GC2, including their surrounding area (approximately 15 m), were made available for this study (Figure S1). The cameras recorded continuously, provided sufficient image quality to identify individual bears even at night, and stored footage for up to 2 weeks. To identify bear visits efficiently, the recordings were systematically screened by reviewing 5‐s segments at 1‐min intervals between 7 pm to 12 am, and 12 am to 6 am. Whenever bear activity was detected, the complete video sequence was saved in 1920 × 1080 MP4 format and analysed in detail. The screening procedure was randomly verified by a second observer.

A single observation was defined as the period from the bear's first appearance within the camera field until its final departure from the monitored area. Temporary movements away from the garbage containers were considered part of the same visit provided the bear remained visible within the camera field and subsequently resumed interaction with the garbage. A new visit was recorded as a new one only after the bear had completely left the camera field and later reappeared.

The saved video recordings were subsequently analysed to quantify several behavioural variables: (1) *Hesitation time* was defined as the interval between the bear's first appearance in the camera field and its entry into the garbage container island. This variable served as a behavioural proxy for the assessment phase preceding resource exploitation; (2) *Vigilance behaviour* was quantified as the cumulative duration of periods during which bears interrupted foraging within the garbage container island to scan the surrounding environment with their heads raised for potential threats; (3) *Foraging duration* represented the cumulative time spent (without vigilance behaviour) actively searching for or consuming food within the garbage container island. If a bear temporarily left the space between the containers but remained visible within the camera field before re‐entering the island, the entire period was considered part of the same foraging event. *Side foraging* was defined as food acquisition from garbage located outside the garbage container islands (e.g., waste bags placed beside the container islands, outside of the electric fences). This behaviour was recorded separately as a distinct behavioural category, because bears might gain potential benefits with foraging outside the garbage islands while avoiding the risks of direct entry into the contained area of the garbage container islands. Additional variables recorded for each visit included: (4) *Total visit duration*; (5) *Garbage availability* outside the containers (1 = visible garbage outside the containers before the bear entered the garbage container island; 0 = no visible garbage at the beginning of the observation); (6) *Electric fence status* (1 = at least one wire activated or 0 = not); (7) *Number of bears* present in each footage (females accompanied by cubs were treated as a single social unit); (8) *Exact date and time* of bear arrival and departure; and (8) *Individual bear ID*, including age and sex class (adult male, adult female, subadult or cub). Individual bears were identified using distinctive morphological characteristics (e.g., body and head shape, open colour patches on the shoulders) at during direct observations at their first appearance in the town. These field observations enabled reliable recognition of individuals in the surveillance footage throughout the study period.

The amount of garbage inside the containers could not be measured or reliably approximated, as the containers were closed and their contents were not visible on the camera footage. Therefore, only the presence or absence of garbage outside the containers, which could be directly observed from the camera footage, was included as a predictor in the analyses. Neither the quantity of garbage outside the containers could be reliably quantified. Accurate estimation would have required direct field measurements, whereas the present study relied exclusively on camera footages.

### 1.3 Statistical Analysis

We examined the durations of bear‐specific behaviours as dependent variables: hesitation, vigilance, and foraging for food. Additionally, we used the total visit duration as a separate dependent variable, encompassing all types of bear activities to represent the overall presence of the observed animals at the study sites. The recordings of both cameras were included in the analyses.

We used linear mixed‐effects modelling (LMM, Gaussian error, maximum likelihood fit) to account for the hierarchical and repeated‐measures structure of the data, with observations nested within individual bears. Behavioural response variables were modelled using Gaussian error structures after appropriate transformations when necessary. In every model, to account for temporal dependence between successive behavioural observations, we fitted continuous‐time autoregressive (CAR(1)) correlation structures within individuals, allowing residual correlations to decay as a function of the irregular time intervals between observations. This approach explicitly accounts for temporal autocorrelation in behavioural sequences and is appropriate for unequally spaced time series data. In the model analysing the duration of hesitation, explanatory variables included the log‐transformed visit order (to account for the potential decrease in effect), the presence of garbage, and the activation status of the electric fence. Based on the timing of consecutive observations, the observations on both camera footage were ordered together, so only bear ID was included as a random factor. We acknowledge that other external factors, such as passing humans and vehicles, influence hesitation, but we did not include them in the statistical analysis due to the low number of instances. Similar models were built for the other three dependent variables (foraging for food, vigilance, and total visit duration), except that only observations without the activated electric fence were included in these analyses.

All statistical analyses were carried out in the R Statistical Environment (R Core Team 2026). LMMs were performed using the *lme* function of the ‘nlme’ package (Pinheiro et al. 2026). If necessary, the variables were log‐transformed before analyses to satisfy the assumptions of normality and homogeneity of variances. Multicollinearity among predictors was assessed using variance inflation factors (*vif*, ‘car’ package; Fox and Weisberg 2019), which indicated no evidence of problematic collinearity (all VIF < 1.1). Model inference was primarily based on effect sizes, confidence intervals, and model comparison using Akaike's Information Criterion (AIC), rather than sole reliance on asymptotic *p*‐values from Wald‐type tests. All models were inspected to ensure adequate convergence and absence of model misspecification using the DHARMa package (Hartig 2024).

## 2 Results

### 2.1 The Use of Electric Fences and the Presence of Garbage at the Two Garbage Islands

At GC1, we observed that the electric fence (at least one of the three wires) was activated 63 (17.26%) days, and at GC2, it was activated 147 (40.27%) days. In contrast, the fence remained inactive for 302 days (82.74%) at GC1 and 218 days (59.73%) at GC2. Monthly patterns revealed clear differences in electric fence use between the two garbage container islands. Overall, the fence was used considerably more frequently and consistently at GC2 than at GC1, whereas activation at GC1 was delayed until May, and even after bears began entering the town in late spring, it remained sporadic thereafter (Figure 2).

**FIGURE 2:**
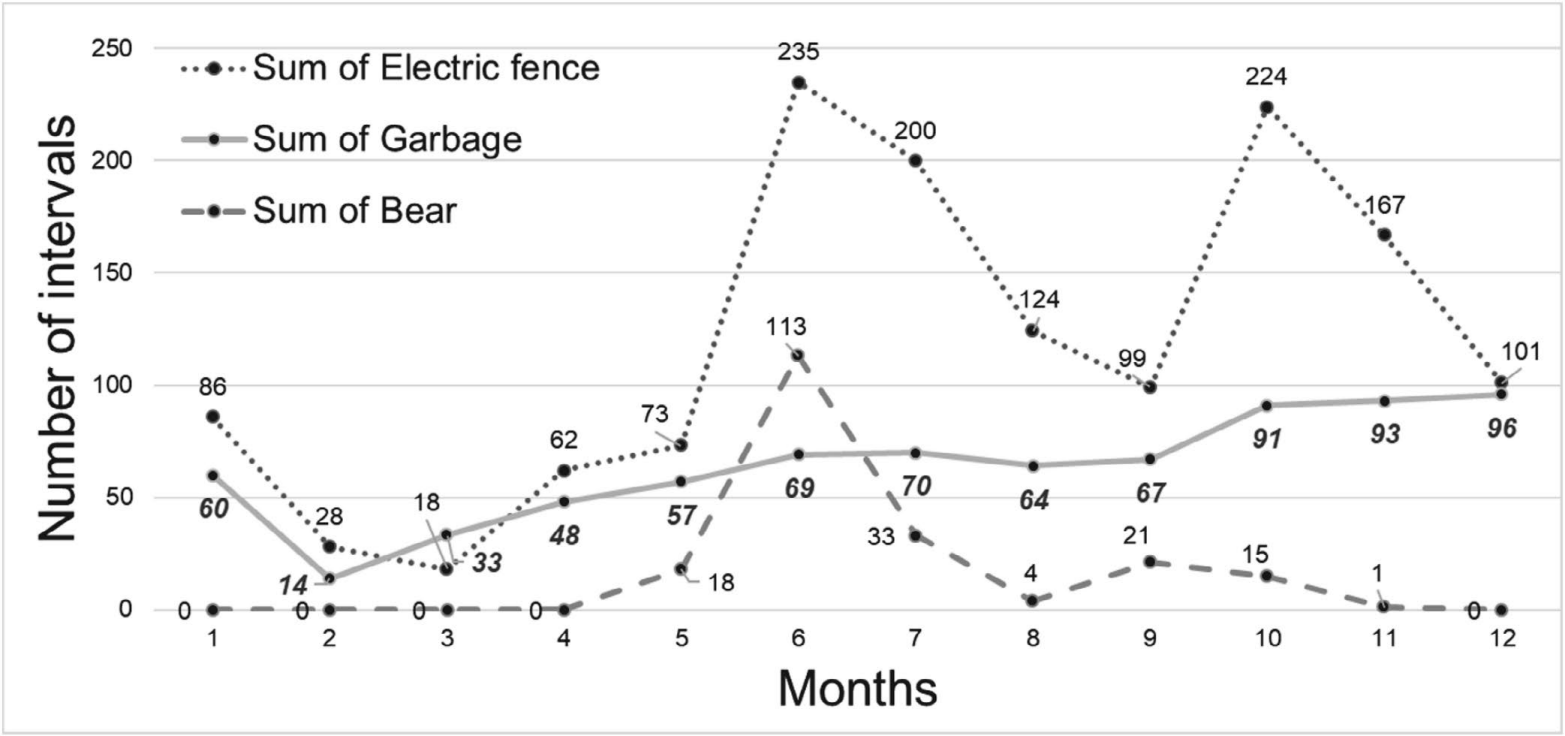
Summed monthly frequencies of electric fence use, visible garbage presence, and bear activity at garbage container islands (GC1 and GC2) during the one‐year study period. Numbers above each line indicate the monthly totals for days with an electric fence, days with visible garbage outside the containers, and recorded bear visits, respectively.

Over 1 year at the GC1, garbage was visible among the containers on 166 (45.47% of the total monitored time) days, while the container surroundings remained empty for 199 (54.53%) days. At GC2, less garbage was observed throughout the year, with visible garbage on 128 (35.06%) days and none present on 237 (64.94%) days. The monthly view at both garbage bin islands revealed that the presence of garbage was higher during the summer months compared to the rest of the year (Figure 2).

### 2.2 The Individual Bears

In this study, we identified all the observed bears individually, a total of 8 individuals: 1 subadult bear, 5 adults (comprised of 1 male and 4 females with cubs), and 2 orphan cubs. The latter two were observed roaming separately from their mother. We found that bears did not use the garbage islands with the same intensity. At GC1, we recorded a total of 179 bear detections: the male was present in 28 cases, and the remaining 151 cases belonged to four different females with cubs. Conversely, at GC2, we recorded 32 bear detections: four observations of a subadult bear, ten observations of a female with cubs that visited GC1 as well, and on 18 occasions the 2 orphan cubs were observed together.

Among the eight individually identified bears, only the two orphan sibling bears were observed visiting a garbage container island simultaneously; there was no temporal overlap among the other six individual bears. The mean (±SD) durations of the recorded bear behaviours, along with the total visit duration for each garbage container island (GC1 and GC2), are presented in Table 1.

**TABLE 1:** The total time brown bears ( *Ursus arctos* ) spent hesitating, being vigilant, foraging among the containers, and the total time spent visiting the garbage containers, with observations recorded separately at the two surveyed garbage container islands (GC1 and GC2). For the complete dataset, see Supporting Information.

Bear observations	Garbage container island1 (GC1) (h/m/s)	Garbage container island2 (GC2) (h/m/s)
Hesitation duration	2: 47:12	0: 10:21
Vigilance duration	2:47:42	0:11:26
Foraging duration	7:19:28	3:14:13
Total visit duration	14:40:09	3:42:32

For two female bears with cubs, we observed that occasionally they left their cubs in a “safe location” instead of bringing them into the more exposed area of the garbage islands. At GC1, females were observed without cubs on 46 occasions, while at GC2 this occurred three times (see Tables S1 and S2 for further details).

During the study, bears at GC1 exhibited a range of behaviours: they left the site with garbage on 65 occasions, showed vigilant behaviour on 29 occasions, interacted with humans on 9 occasions (including 2 fake charges and 7 instances of flight reactions), fled upon encountering moving vehicles on 20 occasions and remained on‐site on 2 occasions. At GC2, bears left with garbage on 1 occasion, exhibited vigilant behaviour on 11 occasions, had no direct contact with humans, fled from moving vehicles on 12 occasions and remained on‐site once.

Overall, bears in our study were predominantly nocturnal, with activity observed primarily between 8 pm and 6 am. The peak activity period was from 12 am to 4 am (Figure 3). For further details regarding the individual bear activity, see the Supporting Information.

**FIGURE 3:**
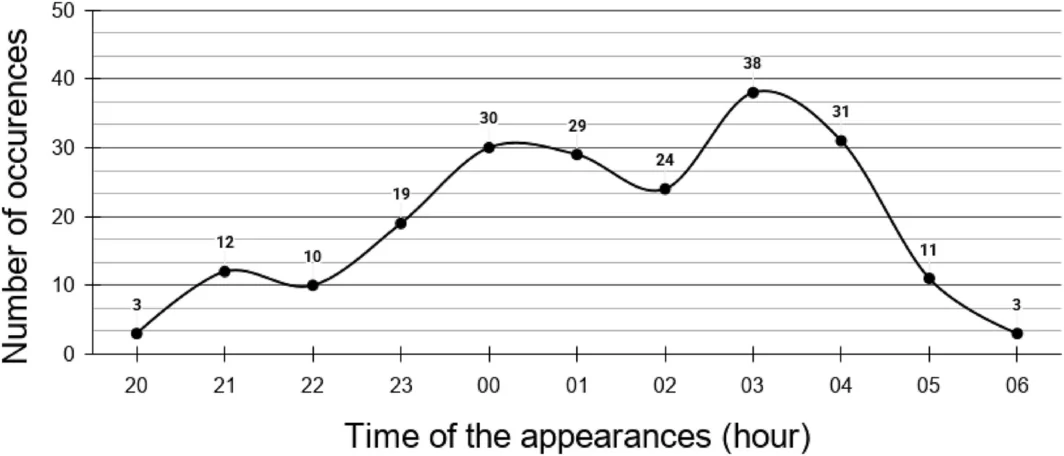
Summed bear activity at garbage container islands 1 (GC1) and 2 (GC2), highlighting the hours of appearance and the corresponding number of occurrences.

### 2.3 Changes in the Behaviour of Bear Individuals

After accounting for repeated observations within individuals and temporal autocorrelation, hesitant behaviour decreased with repeated visits (*β* = −0.443, CI [−0.671 to −0.209]; *χ* ^2^ = 14.101, *p* < 0.001; Figure 4). The installed electric fence increased the duration of hesitation (*β* = 0.646, CI [0.116–1.176]; *χ* ^2^ = 5.785, *p* = 0.016; Figure 5). The estimated temporal autocorrelation parameter indicated moderate positive dependence between successive observations within individuals (*φ* = 0.486, CI [0.251–0.728]). The time spent with vigilance also decreased with repeated visits (*β* = −0.601, CI [−0.922 to −0.279]; *χ* ^2^ = 13.670, *N* = 129, *p* < 0.001; Figure 6), whereas the presence of garbage led to a marginally significant increase in the time spent with vigilance (*β* = 0.766, CI [−0.073 to 1.605]; *χ* ^2^ = 3.271, *N* = 129, *p* = 0.071). If garbage was not present, the bears did not spend time with vigilance. The autocorrelation parameter indicated weak temporal dependence among successive observations of vigilance (*φ* = 0.292, CI [0.076–0.675]).

**FIGURE 4:**
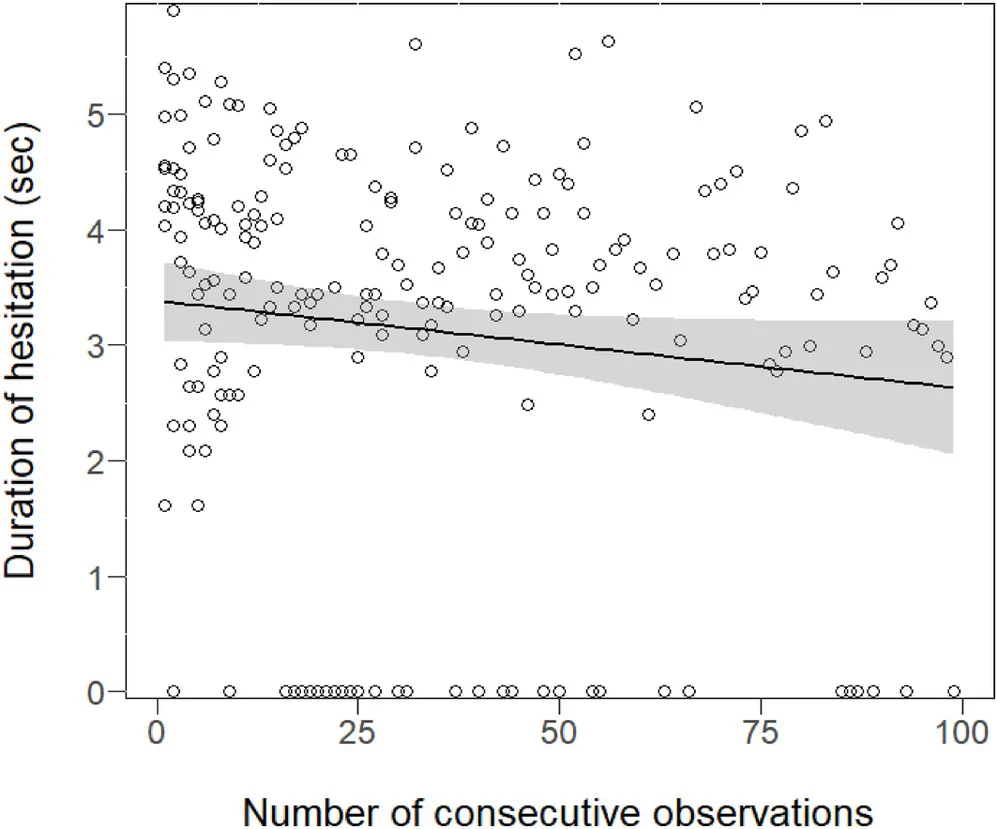
Significant decrease in the duration of hesitation (sec) over the course of consecutive observations.

**FIGURE 5:**
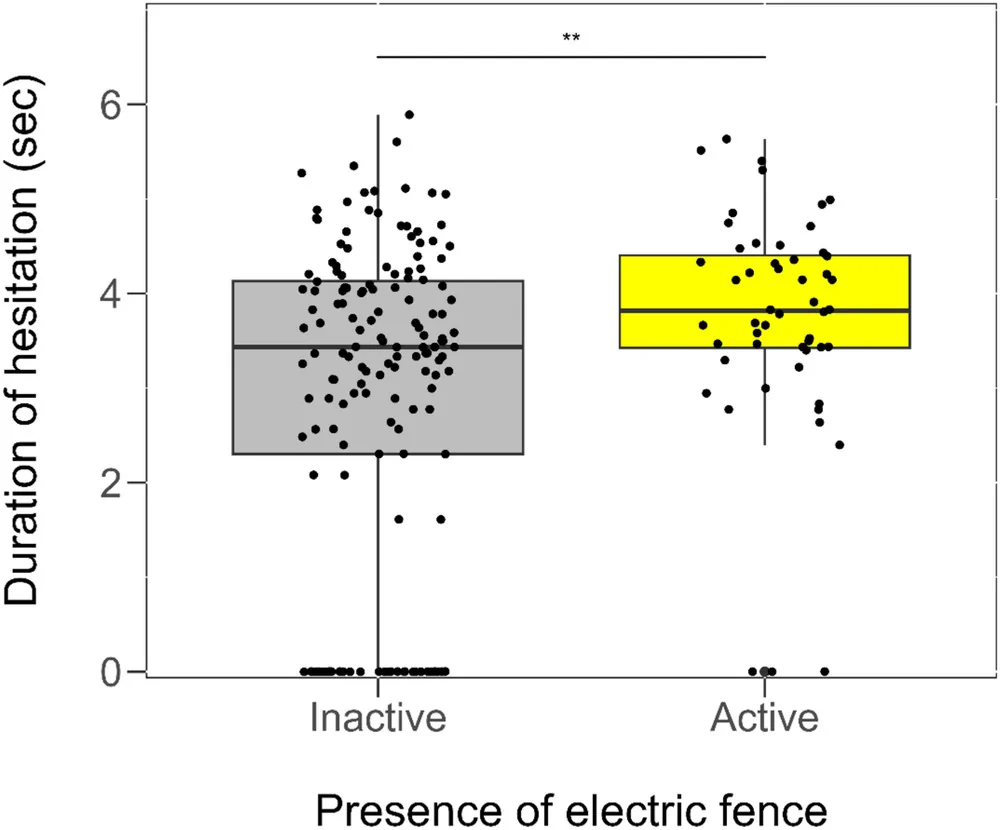
Installed (yellow) electric fence increased the duration of hesitation compared to the situations when the electric fence was not installed (grey).

**FIGURE 6:**
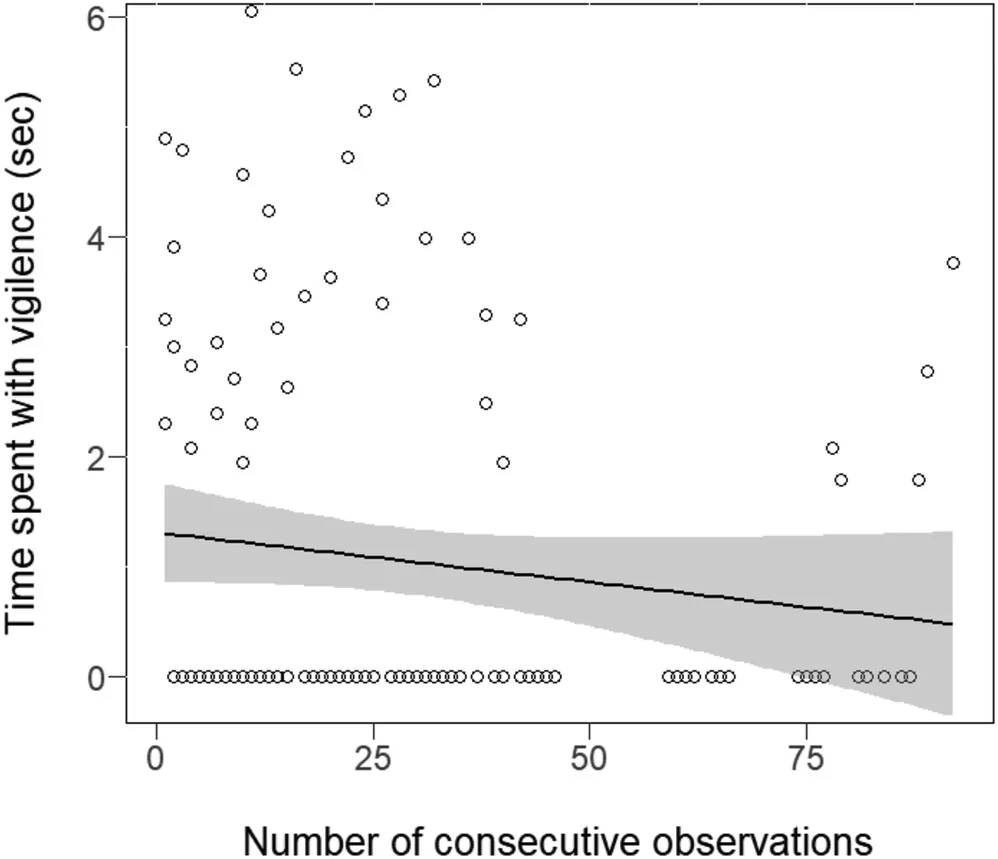
Time spent (sec) with vigilance decreased over the course of consecutive observations.

On the other hand, the time spent foraging for food increased with repeated visits (*β* = 0.505, CI [0.161–0.849]; *χ* ^2^ = 8.410, *N* = 157, *p* = 0.003). The presence of garbage had a strong positive effect on the time spent foraging for food (*β* = 2.634, CI [1.819–3.458]; *χ* ^2^ = 39.878, *N* = 157, *p* < 0.003; Figure 7). Temporal autocorrelation was weak (*φ* = 0.177, CI [0.015–0.756]). The total time spent around the garbage containers, including all types of bear activities, did not increase over the course of consecutive observations (*β* = 0.119, CI [−0.026 to 0.263]; *χ* ^2^ = 2.645, *N* = 157, *p* = 0.104). However, the total time spent around the garbage containers significantly increased when garbage was present (*β* = 0.914, CI [0.526–1.302]; *χ* ^2^ = 21.641, *N* = 157, *p* < 0.001; Figure 8). The estimated temporal autocorrelation indicated moderate dependence between successive observations (*φ* = 0.449, CI [0.208–0.717]). The elevated values in the “absent” column on our figures (Figures 7 and 8) are because our data include all measured activities, including side foraging. For individual variation in behaviour see Supporting Information.

**FIGURE 7:**
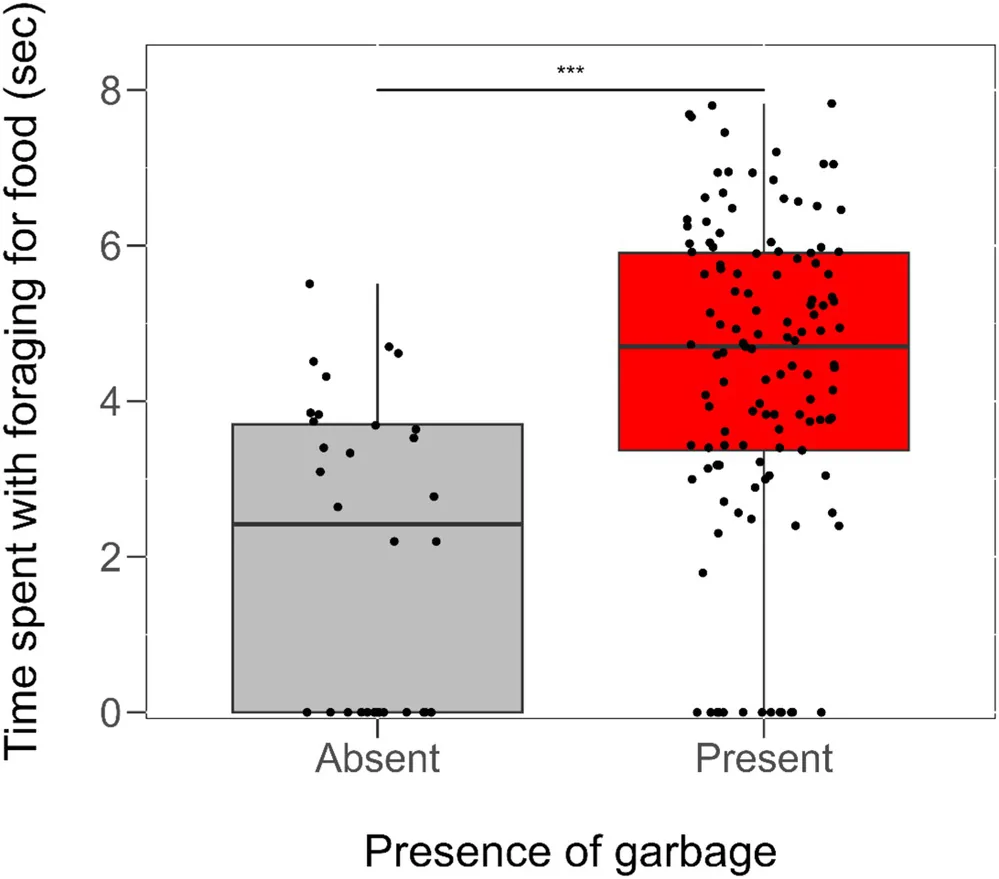
Effect of the presence (red) of garbage on the time spent (sec) with foraging compared to observations when garbage was not present (grey).

**FIGURE 8:**
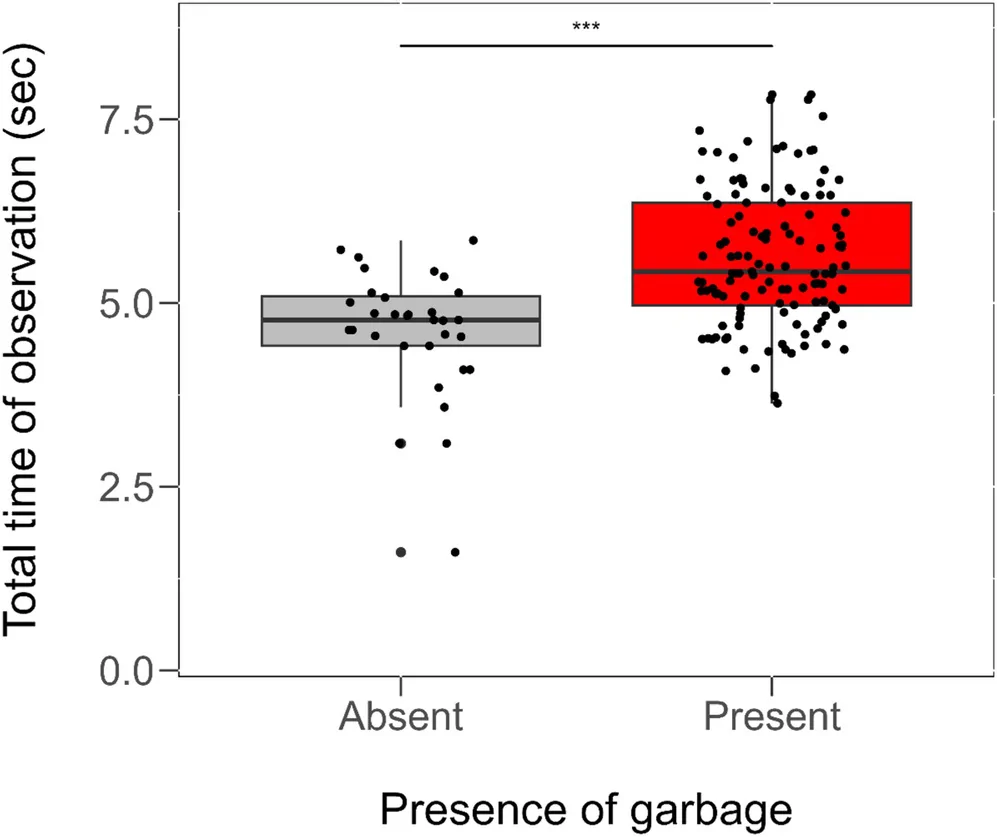
Effect of the presence (red) of garbage on the total time of observation compared to the observations when garbage was not present (grey).

## 3 Discussion

According to our results and in line with our expectations, the duration of hesitation decreased as the number of consecutive observations increased. Thus, hesitation appears to be a useful indicator of increased tolerance, as it likely reflects the time bears spend assessing whether the benefits of foraging for food in the town outweigh the potential risks associated with encountering humans. Reduced hesitation during repeated visits aligns with the definition of habituation formulated in Blumstein (2016), according to which behavioural responses to repeated, non‐threatening stimuli gradually diminish over time. As bears repeatedly encountered this situation without experiencing negative consequences, the direct benefits of reacting fearfully decreased, resulting in progressively shorter hesitation time, consistent with a habituation process.

Considering the effects of the electric fence on hesitation, our results were inconclusive. The electric fence was active during substantially fewer observations than it was inactive. However, the average hesitation time was higher when the electric fence was active, suggesting that bears spent more time assessing whether the protected resource could still be accessed. Only two bears were observed to be directly deterred by the electric fence after making contact with it. In most cases, bears did not approach the fence close enough to touch it, suggesting that the visual presence of the fence or previous experience may have influenced their decision to avoid entering the garbage container island. Our results indicate that using the electric fence as a proactive measure was effective in deterring bear activity (Barrett et al. 2014; Huygens and Hayashi 1999; Khorozyan and Waltert 2020). However, at GC1, the wires were not activated until May, by which time bears were already present in the town. This suggests that people started activating the electric fence only after becoming aware of bear presence in the town. Similarly to the findings of Elfström, Davey, et al. (2014), bear activity in our study was predominantly observed during the summer. This seasonal activity visibly influenced electric fence usage by residents, as the fences were activated more frequently in this period.

In contrast, when we examined the effect of garbage on the time bears spent foraging for food, we found clear evidence that garbage acts as a strong attractant for bears in the town. The total time bears spent in front of the cameras increased when garbage was present in or around the containers. Human food waste, being higher in calories and other essential nutrients than bears' natural food sources, is a convenient and easily accessible food source when adequate protection or storage management is lacking (Elfström, Davey, et al. 2014). Thus, garbage containers in anthropogenic areas become strong attractants for bears, which can travel up to 38 km to reach refuse dumps containing such food sources (Rigg 2005). Therefore, it is not surprising that bear activity at the two garbage container islands was strongly associated with garbage availability. Visible garbage outside the containers was recorded more frequently at GC1 than at GC2. The frequency of visible garbage outside the containers was highest during October–December, whereas bear activity peaked during the summer. This seasonal mismatch likely reflects the bears' natural annual activity cycle, as most individuals entered winter sleep before the period when visible garbage outside the containers was recorded most frequently. Nevertheless, the observed visits to the garbage container islands represented only brief episodes within the bears' annual activity patterns. Bears typically appeared in the town during certain periods, incorporated garbage container islands into their movement and foraging routines while using the urban environment, and then disappeared for days or even weeks before returning. Consequently, our observations should not be interpreted as evidence that the monitored bears relied exclusively on anthropogenic food or abandoned natural food resources.

Besides hesitation, vigilance may also serve as an indicator of increasing tolerance to humans or vehicles (Dyck and Baydack 2004), as the time bears spent to scan their surroundings decreased over consecutive observations. Bears also tended to be more vigilant when visible garbage was present outside the containers at the beginning of the observations. One possible explanation is that the presence of readily accessible garbage increased feeding opportunities while simultaneously exposing bears to greater disturbance or competition, leading them to interrupt foraging more frequently to monitor their surroundings. Over time, however, vigilance and surveying their surroundings declined, suggesting an increasing tolerance of the urban environment and a reduction in initial fearfulness.

The behavioural patterns observed in this study are best interpreted within the broader context of individual behavioural variation. Such variation may arise from differences in behavioural plasticity, previous experience, age, sex and developmental stage, and has been discussed within the framework of animal personality, defined as consistent among‐individual behavioural variation across time and ecological situations (Réale et al. 2007; Sih et al. 2012). Our findings revealed individual differences in the behavioural responses examined. Individuals can vary in their responses to environmental change (Dingemanse and Wolf 2013; Mitchell and Biro 2017). Behaviourally plastic individuals, which can adjust their behaviour according to changing environmental conditions, may gain a fitness advantage over less plastic individuals (Dingemanse and Wolf 2013; Westneat et al. 2014; Mitchell and Biro 2017). The existence of personality has already been demonstrated in black bears ( *Ursus americanus* ) (Myers and Young 2018) and brown bears (Bereczky 2016; Hertel et al. 2019). For example, research on brown bears identified a behavioural syndrome involving activity–exploration and, to some extent, boldness, which affects factors like daily travel distance, preference for nocturnal or diurnal movement, and the selection of or avoidance of open areas such as bogs and forest clearings (Hertel et al. 2019). Comparable behavioural variation was observed among the bears in our study. Behavioural responses differed consistently among identified individuals, indicating that bears did not respond uniformly to the same urban environment. These differences likely reflect the combined effects of previous experience, sex, reproductive status and developmental stage, all of which may influence how individuals perceive and respond to anthropogenic food resources. Consequently, behavioural adjustments associated with food conditioning and habituation should be interpreted as individual‐specific processes rather than uniform responses across the population. This interpretation is illustrated by the case of Pite, which already displayed a high level of tolerance towards the urban environment when first recorded, whereas other individuals showed more gradual behavioural adjustments during the study.

Numerous studies have also confirmed that subadult bears and females, particularly those with cubs, are more commonly observed near settlements than adult males or solitary adult females (Morales‐González et al. 2020). We recorded only one adult male entering the town; all the others were females with cubs and a subadult bear. Consistent with previous studies, bears in our study shifted their activity to nocturnal hours to avoid encounters with humans (Ordiz et al. 2014; Morales‐González et al. 2020; Penteriani et al. 2020). The exception was a very bold female with three cubs, observed several times in different parts of the town during the day, suggesting high tolerance towards human presence. The long‐term effects of growing up in urban environments and learning to exploit human resources remain poorly understood, although maternal behaviour and early‐life exposure may promote the development of increased tolerance in offspring. A study on bears found that males born to habituated mothers are more likely to come into conflict with humans in remote areas (Shimozuru et al. 2020). Nevertheless, prolonged exposure to urban environments during early life may facilitate tolerance and/or habituation later in adulthood, resulting in longer foraging times, reduced vigilance in bold individuals, or continued risk‐avoidance in shyer ones. In our case, Snickers and Bounty, the two orphaned cubs of a female bear (Rozi) that frequented the town for 2 months, learned how to forage on garbage and continued to do so even after their mother was removed (see Supporting Information for further details). Although they were mostly observed together, there were two occasions when one cub was probably outside the cameras' field of view within the safe zone. It is unknown how parental habituation affects offspring survival in bears living in anthropogenic environments, but in general, habituation reduces the fitness of adult females and of their offspring due to the increased risk of human‐caused mortality (Shimozuru et al. 2020). This was evident in the mother bear, as she was removed from the town after her second return. Long‐term monitoring of the cubs following the removal of their mother would represent an interesting avenue for future research.

Although we did not directly study human‐bear interactions, we recorded only nine interactions, during which bears mostly fled upon seeing humans (seven instances). This is consistent with previous studies showing that bears typically avoid confrontation with humans (Moen et al. 2012). On two occasions, bears exhibited defensive reactions in the form of bluff charges (Bombieri et al. 2023), which ended harmlessly without direct physical contact and appeared to serve as warning displays when humans approached within the bear's apparent tolerance threshold (approximately 30 m) without retreating. Bears also generally avoided moving vehicles within approximately 5 m, interacting with a total of 35 vehicles across the two garbage container islands. In 32 cases they fled, whereas they remained at the site during only three encounters. Together, these observations further support the conclusion that the bears in our study generally avoided direct human disturbance.

Our observations also did not support the idea that bears learned the garbage collection schedule, as they continued to visit the container islands even shortly after the containers had been emptied. Although individuals repeatedly exploited these sites, the cumulative time each bear spent foraging there over the one‐year study was relatively limited. Moreover, bears had access to numerous alternative food resources, including natural foods and seasonally abundant anthropogenic resources such as apple and plum trees throughout the town. Consequently, our observations should not be interpreted as evidence that the monitored bears relied exclusively, or even predominantly, on garbage. Rather, they demonstrate that anthropogenic food resources represent predictable, easily accessible supplementary food resources that are repeatedly exploited and may facilitate food conditioning while promoting increasing tolerance towards the urban environment.

The composition and energetic value of anthropogenic food were beyond the scope of the present study and are addressed separately (Gal et al., in press). Accordingly, our objective was not to quantify the relative contribution of anthropogenic food to the bears' annual diet, but to determine whether repeated use of urban garbage is associated with behavioural changes consistent with the early stages of food conditioning and tolerance to human environment.

## 4 Conclusions

Tolerance towards urban environments arises through a complex interplay of ecological and behavioural factors (Bombieri et al. 2021), which may increase through repeated exposure (Ditmer et al. 2019), provided they do not actively avoid humans. However, most bears exhibited increasing tolerance towards the urban environment while still maintaining clear avoidance responses to humans or moving vehicles. According to Blumstein (2016), intermittent exposure to anthropogenic stimuli with long intervals between encounters may not lead to habituation, as true habituation requires repeated and frequent stimulation. None of the monitored bears was exposed frequently enough under comparable conditions to allow the development or demonstration of true habituation as defined by Blumstein (2016). Rozi, the female bear that was eventually removed, illustrates this distinction well. Although she had repeatedly visited the town since 2018 (the first video recording in which she can be identified), she continued to flee from approaching people despite regularly exploiting garbage container islands. Thus, repeated use of anthropogenic food resources alone did not eliminate avoidance behaviour towards humans. In contrast, environments that provide frequent, predictable human‐bear interactions, such as roadside feeding sites, may promote stronger habituation because the behavioural stimulus is more consistent. In our study, bears repeatedly returned to garbage container islands and showed increasing tolerance towards the urban environment while continuing to avoid direct encounters with humans. Our findings therefore highlight that effective garbage management and the consistent use of preventive measures, particularly properly maintained electric fences, are key management actions for reducing repeated bear visits and limiting the behavioural changes that may ultimately lead to food conditioning and, under continued exposure, increased tolerance or even habituation to urban environments.

## Author Contributions

**László Gál:** conceptualization (lead), data curation (lead), formal analysis (equal), investigation (lead), methodology (equal), project administration (lead), supervision (equal), validation (lead), visualization (equal), writing – original draft (supporting), writing – review and editing (equal). **Írisz‐Barbara Dobos:** conceptualization (supporting), data curation (equal), formal analysis (supporting), methodology (equal), writing – original draft (lead). **András Liker:** formal analysis (supporting), supervision (supporting), writing – review and editing (equal). **István Elek Maák:** formal analysis (lead), methodology (supporting), software (lead), supervision (equal), visualization (equal), writing – review and editing (equal).

## Funding

This work was supported by the Ministry of Research, Innovation and Digitisation, CNCS/CCCDI – UEFISCDI, 25/2024 COFUND‐BIODIVMON‐MonitAnt. Hungarian Academy of Sciences, BO/00708/22. HUN‐REN TKI Hungarian Research Network, 1600707.

## Ethics Statement

In our work, as we used camera footage to assess bear behaviour, we did not harm them in any way, and no special approvals were required, aside from the city council's agreement to use the surveillance recordings.

## Conflicts of Interest

The authors declare no conflicts of interest.

## Supporting information


**Data S1:** Supporting Information.

**Figure S1:** Views of garbage container islands GC1 and GC2 from the perspective of the surveillance cameras. The figure also indicates the safe zones (shown in green areas with trees), which provided bears with sheltered locations where they could consume anthropogenic food.
**Figure S2:** The daily activity periods (hours) of Bonbon, Fánk, Igor, Pite and Rozi at GC1 island, with the number of occurrences shown above each time point. Different point shapes represent different individuals.
**Figure S3:** The daily activity periods (hours) of Berkenye, Bounty, Snickers and Rozi at GC2 island, with the number of occurrences shown above each time point. Different point shapes represent different individuals.
**Table S1:** The time Bonbon, Fánk, Igor, Pite and Rozi spent (measured in hours, minutes and seconds) with different behaviours at the GB1 island. Total time of presence (all presence) and total time (all activity) spent with different activities (hesitation, feeding on garbage, feeding outside) is shown. In female bears, the number of cubs is shown in parentheses.
**Table S2:** The time Berkenye, Bounty, Snickers and Rozi spent (measured in hours minutes and seconds) with different behaviours at the GC2 island. Total time of presence (all presence) and total time (all activity) spent with different activities (hesitation, feeding on garbage, feeding outside) is shown. In Rozi, the number of cubs is shown in parentheses.

## Data Availability

All the required data are uploaded as Supporting Information.
