# Supplementary material for: Human Tolerance in Romanian Brown Bears (Ursus arctos): Access to Human Food Sources Can Lead to Changes in Bear Behaviour
Source: Ecol Evol. 2026 Nov 9;16(11):e74359. doi: 10.1002/ece3.74359 (PMC13746096; doi:10.1002/ece3.74359)
Supplement: Supplementary file 1 [file ECE3-16-e74359-s002.docx]

**Supplementary Material**

**Title:** **Human tolerance in Romanian brown bears (*Ursus arctos*): Access to human food sources can lead to changes in bear behaviour**

László Gál, Hungarian Department of Biology and Ecology, Babeş-Bolyai University, Clinicilor 5–7, 400006 Cluj-Napoca, Romania; Laboratory for Sociobiology and Insect Ecology, Centre for Systems Biology, Biodiversity and Bioresources, Faculty of Biology and Geology, Babeș-Bolyai University, Clinicilor 5–7, Cluj-Napoca, Romania

Írisz-Barbara Dobos, Hungarian Department of Biology and Ecology, Faculty of Biology and Geology, Babeș-Bolyai University, Clinicilor 5–7, 400006 Cluj-Napoca, Romania

András Liker, HUN-REN-PE Evolutionary Ecology Research Group, University of Pannonia, Veszprém, Hungary; Behavioral Ecology Research Group, Center for Natural Sciences, University of Pannonia, Veszprém, Hungary

István Elek Maák, Department of Ecology, University of Szeged, Közép fasor 52, 6726 Szeged, Hungary; Museum and Institute of Zoology, Polish Academy of Sciences, Twarda 51-55, 00-818 Warsaw, Poland

Correspondence: László Gál, Hungarian Department of Biology and Ecology, Babeş-Bolyai University, Clinicilor 5–7, 400006 Cluj-Napoca, Romania. Email: laszlo.gal@ubbcluj.ro

**Fig. S1.** Views of garbage container islands GC1 and GC2 from the perspective of the surveillance cameras. The figure also indicates the safe zones (shown in green areas with trees), which provided bears with sheltered locations where they could consume anthropogenic food.

### **Individual bears at the GC1 garbage bin island**

“Bonbon”, a female with three cubs, first appeared on 9 September 2022, and was last seen on 12 September, during which time it visited GC1 a total of 55 times mainly between 3 a.m. and 5 a.m. (Fig.S1). Over this period, Bonbon was present at GC1 for a total of 4 hours 16 minutes and 48 seconds, and spent this time feeding on garbage inside the bins, foraging in the open area outside the bins and hesitating (Table S1). On one occasion, Bonbon fled after encountering a human, and on two occasions, she fled upon seeing moving vehicles. It showed vigilant behaviour 7 times and left the site with garbage 27 times. Bonbon’s cubs were present with her 27 times, of which both cubs 23 times and only one 4 times. The cubs were not seen 28 times. There was only one time when they got in direct contact with garbage inside the garbage bin island. When not present, we believe they may have been left in a safe zone, possibly in the area above the garbage bins. This inference is based on the fact that Bonbon often carried garbage in that direction when the cubs were not visible, presumably bringing food to them.

**Fig. S2.** The daily activity periods (hours) of Bonbon, Fánk, Igor, Pite and Rozi at GC1 island, with the number of occurrences shown above each time point. Different point shapes represent different individuals.

### “Fánk”, another female with 2 cubs, visited GC1 island twice during the same night (20 June 2022) when the electric fence was used (between 12 a.m. and 1 a.m.; Fig. S1). Fánk was present for a total of 5 minutes and 58 seconds, but during this time we observed no feeding on garbage inside the bins or in the open area outside the bins, and spent only 3 minutes 44 seconds hesitating (Table S1). The cubs managed to make contact with the garbage after jumping over the lowest wire. When Fánk attempted to follow them, it was shocked by the fence, which suggests that she had not previously encountered electric fences and was unacquainted with their deterrent effect. She never got in contact with humans or moving cars.

The male bear we named “Igor” first appeared on 02 October 2021 and was last seen on 15 October, during which time it visited GC1 a total of 28 times. Igor’s primary activity occurred between 11 p.m. and 12 a.m. (Fig. S1). Over this period, Igor spent a total of 1 hour, 57 minutes, and 42 seconds at GC1 with foraging inside the bins, foraging in the open area around the bins, and hesitating (Table S1). Igor exhibited vigilant behaviour once and left the site carrying garbage 11 times.

**Table S1.** The time Bonbon, Fánk, Igor, Pite and Rozi spent (measured in hours, minutes and seconds) with different behaviours at the GB1 island. Total time of presence (all presence) and total time (all activity) spent with different activities (hesitation, feeding on garbage, feeding outside) is shown. In female bears, the number of cubs is shown in parentheses.

| **ID** | **All presence** | **All activity** | **Hesitation** | **Feeding on garbage** | **Feeding outside** |
| --- | --- | --- | --- | --- | --- |
| Bonbon (♀; 3) | 04:16:48 | 03:51:09 | 00:54:25 | 02:12:07 | 00:44:37 |
| Fánk (♀; 2) | 00:05:58 | 00:03:44 | 00:03:44 | 00:00:00 | 00:00:00 |
| Igor (♂) | 01:57:42 | 01:43:02 | 00:20:58 | 01:05:34 | 00:16:30 |
| Pite (♀; 3) | 00:09:30 | 00:06:52 | 00:006:52 | 00:00:00 | 00:00:00 |
| Rozi (♀; 3) | 08:10:11 | 05:59:40 | 01:21:23 | 04:01:47 | 00:36:40 |

Igor was a rare case of a male bear entering a town, spending two weeks at the GC1 island. The approximately 21 min of total hesitation suggests that Igor was a relatively bold individual, it hesitated longer only due to the presence of the electric fence. In all four cases, the electric fence deterred him from entering, suggesting prior experience with such fences. During two encounters with humans, Igor stayed on site once, performing a false charge when a woman stopped to observe him. On another occasion, he fled upon seeing two approaching humans. These two encounters occurred within five minutes of each other, and the false charge happened early in Igor’s feeding, suggesting that the presence of food between the garbage bins might have made him more willing to take the risk of staying and scaring the women away. However, when two more people appeared minutes later, Igor either felt satisfied with its feeding or was deterred by the increased number of humans, leading it to flee. In both encounters with moving vehicles, Igor fled immediately.

“Pite”, also a female with three cubs, first appeared on 26 June 2022 and was last seen on 6 July, during which time it visited GC1 a total of five times. Pite’s primary activity occurred between 9 p.m. and 1 a.m. (Fig. S1). Over this period, Pite spent a total of 9 minutes and 30 seconds, but besides hesitating, it spent no time with feeding on garbage inside the bins or foraging in the open area outside the bins (Table S1). Since we observed no feeding time inside the island, Pite never showed vigilant behaviour and never left the site with garbage. In every instance, all three cubs were present with Pite but they never got in direct contact with garbage inside the garbage bin island. During the three encounters with humans Pite never fled, instead stayed on site and false charged towards a woman and a man who were photographing it. While this behaviour might appear threatening, it did not seem to be aggressive. However, when encountering a moving car, it fled. Furthermore, Pite was observed in the town several times during the day, suggesting that it might have been in some stage of habituation, displaying reduced wariness toward humans.

“Rozi”, another female bear with three cubs, stayed in the town for the longest time period, around two months, during which she appeared almost daily at the GC1 island. Rozi first appeared on 5 May 2022, was relocated around 7 July, reappeared on 28 July with a missing cub, and was last seen on July 29, when it was relocated again but the two remaining cubs escaped. Over this time, Rozi visited the GC1 a total of 89 times (Fig. S1). The cubs were also present 71 times, of which all three cubs 53 times, two cubs 12 times and one cub six times. The cubs were not present 18 times. Rozi spent a total of 8 hours 10 minutes and 11 seconds at GC1 mainly with feeding on garbage inside the bins, foraging in the open area outside the bins and hesitating (Table S1). Rozi showed vigilant behaviour 47 times and left the site with garbage 27 times. In her only encounter with humans, Rozi did not flee but was likely not directly approached—she was merely illuminated by a flashlight. Rozi also showed avoidance behaviour toward vehicles, as from the 15 occasions when it encountered moving cars, it fled 14 times, although remained on-site when two stationary cars were present near the garbage bins. It is worth mentioning, that in this latter case, the cars were stationary at the approach of the bins and only one of them moved a few metres while it was still inside the island. Rozi did not flee, but shortly after left with garbage along with her cubs. In general, during the first observations, she was extremely cautious, allowing her cubs to approach the garbage first. Over time, her hesitation decreased, but she continued to exhibit significant vigilance. Interestingly, although Rozi has been observed in the town in 2020, it still exhibited cautious behaviour in 2021, suggesting that prior experience did not entirely eliminate her wariness. Consistent with findings from other studies (Penteriani et al., 2020a), Rozi was primarily nocturnal, with most of her activity occurring after 1 a.m. (Fig. S1), suggesting an attempt to avoid human encounters. Rozi never entered the town during the day. Rozi’s cubs had ample opportunity to learn how to forage in the town, encountering garbage on 32 occasions at GC1.

### **Individual bears at the GC2 garbage bin island**

“Berkenye”, a subadult bear, appeared four times at GC2 on 21 November 2021 between 8 p.m. and 12 a.m. (Fig. S2). Over this period, Berkenye spent time feeding on garbage during the first three visits (total time: 17 minutes and 30 seconds; Table S2). However, during the fourth visit, the electric fence was activated, and Berkenye left without attempting to bypass it, despite knowing there was food inside. This behaviour might indicate prior experience with electric fences, as Berkenye did not return to the site afterward. Berkenye never encountered humans, and when it encountered moving cars, it remained on site once and fled the second time. Berkenye displayed vigilant behaviour twice and never left the site with garbage.

**Fig. S3.** The daily activity periods (hours) of Berkenye, Bounty, Snickers and Rozi at GC2 island, with the number of occurrences shown above each time point. Different point shapes represent different individuals.

“Rozi” also appeared at GC2 10 times, primarily around 4 p.m. (Fig. S2). Rozi was present here for a total for 16 minutes and 42 seconds. During this time, Rozi fed on garbage inside the bins and hesitated but spent no time foraging in the open area outside the bins (Table S2). Rozi did not encounter humans and fled upon seeing a moving car. Rozi displayed vigilant behaviour five times and never left the site with garbage. Rozi’s cubs were also present at GC2 seven times, with all three cubs present six times and one cub present one time. The cubs were not observed three times and were observed with garbage inside the garbage bin island only once. However, the experience at GC2, combined with their time at GC1, likely equipped the remaining cubs with the skills to forage independently after their mother was removed.

**Table S2.** The time Berkenye, Bounty, Snickers and Rozi spent (measured in hours minutes and seconds) with different behaviours at the GC2 island. Total time of presence (all presence) and total time (all activity) spent with different activities (hesitation, feeding on garbage, feeding outside) is shown. In Rozi, the number of cubs is shown in parentheses.

| **ID** | **All presence** | **All activity** | **Hesitation** | **Feeding on garbage** | **Feeding outside** |
| --- | --- | --- | --- | --- | --- |
| Berkenye | 00:17:30 | 00:15:07 | 00:01:44 | 00:13:23 | 00:00:00 |
| Bounty | 01:34:08 | 01:29:41 | 00:02:37 | 01:27:04 | 00:00:00 |
| Snickers | 01:34:12 | 01:27:10 | 00:02:00 | 01:25:10 | 00:00:00 |
| Rozi (♀; 3) | 00:16:42 | 00:12:36 | 00:04:00 | 00:08:36 | 00:00:00 |

“Bounty” and “Snickers” were Rozi’s cubs, and they fled when their mother was removed but reappeared in the town without Rozi after a month. In the town, the two cubs continued their learned behaviour and frequented GC2 from 26 August 2022 until 18 September (Fig. S2). Snickers appeared 10 times, spending a total of 1 hour 34 minutes and 12 seconds feeding on garbage inside the bins, and hesitating, but did not forage in the open area outside the bins (Table S2). Snickers never encountered humans, and in five instances when it encountered moving cars, it fled each time. Snickers showed vigilant behaviour eight times and left the site with garbage once. Bounty appeared eight times and spent a total 1 hour 34 minutes and 08 seconds feeding on garbage inside the bins and hesitating, but did not forage in the open area outside the bins (Table S2). Bounty never encountered humans, and in the five instances when it encountered moving cars, it fled each time. Bounty showed vigilant behaviour nine times and never left the site with garbage. Both cubs appeared together, except from two occasions when Snickers was present alone but did not leave the safe zone to approach the garbage island.
